# Sleep duration, mortality and the influence of age

**DOI:** 10.1007/s10654-017-0297-0

**Published:** 2017-08-30

**Authors:** Torbjörn Åkerstedt, Francesca Ghilotti, Alessandra Grotta, Andrea Bellavia, Ylva Trolle Lagerros, Rino Bellocco

**Affiliations:** 1 Department of Clinical Neuroscience, Karolinska Institutet, 17177 Stockholm, Sweden; 2 Stress Research Institute, Stockholm University, Stockholm, Sweden; 3 Department of Statistics and Quantitative Methods, University of Milano-Bicocca, Milan, Italy; 4 Department of Medicine, Clinical Epidemiology Unit, Karolinska Institutet, Stockholm, Sweden; 5 Department of Medical Epidemiology and Biostatistics, Karolinska Institutet, Stockholm, Sweden; 6 Center for Health Equity Studies, Stockholm University and Karolinska Institutet, Stockholm, Sweden; 7 Institute of Environmental Medicine, Karolinska Institutet, Stockholm, Sweden; 8 Department of Medicine, Clinic of Endocrinology, Metabolism and Diabetes, Karolinska University Hospital Huddinge, Stockholm, Sweden

**Keywords:** Health, CVD, Cancer, Survival analysis, Aging

## Abstract

Prior work has shown that both short and long sleep predict mortality. However, sleep duration decreases with age and this may affect the relationship of sleep duration with mortality. The purpose of the present study was to assess whether the association between sleep duration and mortality varies with age. Prospective cohort study. 43,863 individuals (64% women), recruited in September 1997 during the Swedish National March and followed through record-linkages for 13 years. Sleep duration was self-reported and measured using the Karolinska Sleep Questionnaire, and grouped into 4 categories: ≤5, 6, 7 (reference) and ≥8 h. Up to 2010 3548 deaths occurred. Multivariable Cox proportional hazards regression models with attained age as time scale were fitted to estimate mortality rate ratios. Among individuals <65 years, short (≤5 h) and long (≥8 h) sleep duration showed a significant relationship with mortality (HR 1.37, 95% CI 1.09–1.71, and HR 1.27, 95% CI 1.08–1.48). Among individuals 65 years or older, no relationships between sleep duration and mortality were observed. The effect of short and long sleep duration on mortality was highest among young individuals and decreased with increasing age. The results suggest that age plays an important role in the relationship between sleep duration and mortality.

**Electronic supplementary material:**

The online version of this article (doi:10.1007/s10654-017-0297-0) contains supplementary material, which is available to authorized users.

## Introduction

One of the most common questions concerning sleep is: “how much should we sleep?” Several systematic reviews on sleep duration and mortality have found that the optimal sleep duration is 7 h, with a significant increase of overall mortality for individuals with short (≤6 h) or long (>8 h) sleep [1–3]. In a critical review Kurina et al. [4] argue that results from the literature are not totally consistent. The analysis of Kurina et al. [4] included 42 studies, giving rise to 55 different estimates of the association between sleep duration and overall mortality, based on which only 14 studies (25%) showed a U-shaped relationship, while 23 studies (43%) showed no association at all, and the remainder showed associations for either short or long sleep. Their main arguments against the presence of a U-shaped relationship between sleep and mortality are the heterogeneity in the measurement of sleep duration and in its categorization, the use of different methods and variables for adjusting for potential confounders across studies, and different age ranges.

Since sleep duration decreases with age [5], age may be a confounder of particular interest. Indeed, most studies adjust for it, but the role of age as potential effect modifier has not been explicitly studied other than in a few studies [6]. Thus, Yeo et al. [7] and Gangwish et al. [8] reported that individuals older than, or equal to 60 years, had a U-shaped relationship between sleep duration and mortality, but the same pattern could not be found in those below the age of 60. Hublin et al. [9] found a similar U-shaped pattern for men aged 55 years or older, but this was not seen in women in the same age group. For men younger than 55 years only short sleep was significantly associated with mortality. Two meta-analyses have investigated the effect of age on the relationship between sleep duration and mortality. Cappuccio et al. [1] divided studies into those based on old (≥60 years) or young (<60 years) cohorts, and demonstrated a U-shaped relationship between sleep duration and mortality in both types of cohorts. Liu et al. [10] used 65 year as cut-off to categorize the included studies and showed a significant association between both short and long sleep and the risk of overall mortality among subjects <65 years. Only long sleep duration was associated with an increased risk in participants ≥65. It is worth noting that none of the studies included stratification on age. As a consequence, there is a need for a systematic investigation of the role of age in the relationship between sleep duration and mortality.

The results from the meta-analyses seem to indicate that the U-shaped relationship may be found in all age groups, but nothing is known about more detailed effects of age. Most of the reported original studies are small, and it is not entirely clear why the 60-year cut-off is the most frequently used. One particular problem is that the age group above the age of 60 years will include a mixture of retirees and individuals still in the workforce. Retirement may lead to a different sleep pattern, since there is a possibility for “sleeping in” and nap during weekdays. Possibly, the 65-year cut-off (based on the common retirement age) would provide more homogeneous age groups in terms of sleep patterns.

Daytime napping will increase the total amount of sleep during a 24 h period, and should therefore be of interest when studying effects of sleep duration. Among the few available studies, Lan et al. [11] adjusted for nap duration, which did not affect the results on sleep duration and mortality. Cohen-Mansfield et al. [12] found that 9 h sleep plus napping was associated with increased mortality. The meta-analysis of Liu et al. [10] found a pronounced J-shaped relationship between 24 h sleep duration (including naps) and all cause mortality. However, when night sleep (without naps) was considered, an increased risk was observed only for long sleep. Thus, napping may be an important variable in studies of sleep duration and mortality.

The main purpose of the present study was to quantify the relationship between sleep duration and subsequent mortality, while stratifying on age according to the retirement age cut-off. In addition, we studied how the relationship between sleep duration and mortality was affected by age, in a time dependent analysis.

## Methods

### Study population

In September 1997 a fund-raising event organized by the Swedish Cancer Society (“the Swedish National March”) took place on 3600 sites throughout the country. Participants were asked to fill out a 36-page questionnaire concerning lifestyle and medical history [13, 14]. The study was approved by the Regional Ethical Review Board of Karolinska Institutet, and all subjects provided informed consent. A total of 43,880 individuals returned the questionnaire. We excluded participants with clearly inconsistent answers (n = 4) and subjects who reported an incorrect national registration number (n = 13). Out of the remaining 43,863 subjects we excluded those with missing data on age (n = 11), had an age <18 (n = 1740), or who had emigrated (n = 457), or died (n = 8) before the start of follow-up. We also excluded individuals with missing values on sleep duration (n = 2456). The final analysis included 14,024 men and 25,167 women for a total of 39,191 subjects.

### Exposure assessment

The Karolinska Sleep Questionnaire (KSQ) was used to assess sleep characteristics [15]. The questions included the following: “How many hours, approximately, per night do you usually sleep during a weekday night?” The initial response alternatives were: <5, 5, 6, 7, 8, or ≥9 h. Because of insufficient number of participants in the shortest and longest sleep categories the two lowest and two highest categories, were collapsed. This yielded 4 categories: ≤5, 6, 7 (reference) and ≥8 h, with the extremes reflecting short and long sleep. The reference category was set to 7 h, since that was the most used reference in the meta-analysis by Cappuccio et al. [1], as well as being the reference category in the largest study on sleep duration and mortality, in which more than one million participants were included [16]. In addition, in this large study the observed difference in mortality between 7 and 8 h was significant.

### Case ascertainment

All-cause mortality data were obtained through linkage, using the Swedish National registration number, to the Swedish Cause of Death Register held by the National Board for Health and Welfare. A total of 3548 deaths (1940 men and 1608 women) occurred during 13 years of follow-up (September 1997–December 2010). Main causes of death were cardiovascular diseases (CVD-ICD-10 codes I00-I99) (n = 1140) and cancer (ICD-10 codes C00-C97) (n = 1645).

### Statistical analysis

Baseline characteristics of the study cohort were described according to the four categories of sleep duration. The distribution of all variables, except age and sex, was directly standardized to the age distribution of the entire study population. Differences in baseline variables across categories of sleep duration were statistically assessed using the Student t-test for continuous variables and the Chi square test for categorical variables.

Based on our a priori assumptions on the relationships between confounders, intermediate variables, exposure, and outcome variables, we were able to draw a directed acyclic graph (DAG) [17], with the aim to identify the minimal set of variables to adjust for the total effect (presented as supplementary material). To draw the DAG, the web-based application DAGitty (http://www.dagitty.net) was used [18].

The association between sleep duration and mortality (overall, CVD mortality and cancer mortality) was assessed through a Cox proportional hazards regression model with attained age as time scale. To estimate hazard ratios (HR) and 95% confidence intervals (CI) age-adjusted and multivariable adjusted Cox models were computed. In the multivariable analyses we first controlled for the following potential confounders: sex, body mass index (BMI) (≤25, 25–30, ≥30 kg/m^2^), level of education (9, 11–14, ≥15 years, other), smoking status (never, former, current), alcohol consumption (quintiles of alcohol g/month: <23, 23–124, 124–263, 263–503, ≥503), total physical activity (low ≤34.3, medium 34.3–46.4, high ≥46.4 MET * hours/day, where MET stands for Metabolic Energy Turnover [19]) and major diseases (CVD or cancer diagnosis before October 1997, obtained from the Swedish Patient Register). In addition, two more variables suggested by the DAG were considered, namely occupational status (employed, unemployed, retired, sick leave, other) and depression, based on the combination of the following five variables: feel sad, feel lonely, feel worried, feel unsatisfied, feel unhealthy (never, sometimes, often, always), each reclassified into two groups, often or always indicating presence of the condition; depression was considered present when at least three out of the five conditions were present. However, these two variables were not included due to a large proportion of missingness and degree of potential misclassification and included only in the sensitivity analysis.

We performed stratified analyses (together with a formal test for interaction) to assess the role of sleep duration within two different age groups separated at the retirement age in Sweden (<65, ≥65). In order to investigate whether napping would change the relationship between sleep duration and mortality, the analyses were also stratified by napping (“never”, “seldom” or “sometimes” vs. “most of the time” or “always”).

The proportional hazards (PH) assumption of the covariates fitted in Cox’s regression was tested using both Martingale’s and Schoenfeld’s residuals. Stratified Cox models were used to adjust for variables that did not meet the PH assumption, while predictors for which the PH assumption was satisfied were controlled by including them in the model.

Due to departures from the underlying proportional hazards assumption in the exposure of interest, a simple graphical tool based on restricted cubic splines [20] was applied and graphs of the time-varying hazard ratio for short and long sleep are reported. The time-dependent coefficients for short and long sleep were modelled using restricted cubic splines with 3 knots placed according to Harrell’s recommended percentiles (10th, 50th, and 90th of the distribution of the uncensored event times) [21].

The proportional hazard assumption was violated only by the “major diseases” predictor, and therefore stratified Cox models were fitted throughout the study.

To explore the time dimension of the association between sleeping and mortality we used Laplace regression. This technique allows estimations of the differences in age at death, as a function of the covariates of interest, for each relevant percentile of the age distribution [22]. We applied Laplace regression to estimate the 25th, 50th, and 75th percentiles of age at death, and we adjusted for the same set of confounders as in our main analysis. The Laplace models were further adjusted for baseline age, which was the time scale in the Cox models.

Subjects dying in the first 2 years of follow-up might have been sicker with undiagnosed diseases at the start of follow-up. To deal with this issue of reverse causality we performed a sensitivity analysis excluding the first 2 years of follow-up. Furthermore, we performed sensitivity analyses by additionally adjusting our model for occupational status, depression, snoring; moreover, we also adjusted by self-reported sleep medication use and napping which were considered as mediators in the proposed DAG. As sensitivity analysis, we drew a secondary DAG (graph not shown), including sleep quality and sleepiness. Both variables turned out to be part of the minimal set of covariates we needed to adjust for in order to estimate the total effect of sleep duration on mortality. These were thus added in the main model.

Sleep quality was defined as “good” or “rather good” versus “neither good nor bad”, “rather bad” or “bad”; while sleepiness was defined as being “mostly” or “always” sleepy during the day versus “sometimes”, “seldom” or “never” sleepy.

Lacking a consensus on what should be a proper reference category, we also used 7–8 h as reference, since that was the second most common reference value in the meta-analysis of Cappuccio et al. [1]. The age cut-point of 65 years was based on the common retirement age in Sweden. However, since the present work focuses on age effects we also explored a very low cut-point (45 years) and a higher cut-point (70 years). In addition, we also applied a cut-point of 60 years since most of the few original studies that have analyzed age effects have used 60 years.

Analyses were performed using Stata, version 14 (StataCorp LP, College Station, Texas). All statistical tests were two-sided, and *p* values less than 0.05 were considered statistically significant.

### Missing data

We first studied the distribution of age, sex, BMI, educational level, smoking, alcohol consumption, physical activity, and presence of major diseases among individuals with (n = 39,191) and without (n = 2456) exposure data (sleep duration during working week).

In order to study if exclusion of subjects with missing values would have resulted in overestimation of the true survival of the cohort, we estimated survival curves in subjects with and without missing exposure. Survival curves were adjusted for the before mentioned variables and were obtained through flexible parametric survival models, with follow-up time as time-scale [23].

The proportion of missing values was 14.9% for occupational status, 7.7% for smoking status, 6.4% for physical activity, 4.1% for BMI, 1% for educational level and less than 1% for alcohol consumption, napping, snoring, hypnotics and depression. We assumed that data were missing at random (MAR). Multiple Imputation Chained Equation (MICE) with 20 imputations was used to impute missing data on covariates [24]. The variables in the model were the same as the ones appearing in the complete-data model including information on survival outcomes (Nelson-Aalen cumulative hazard and mortality status).

## Results

**Fig. 1:**
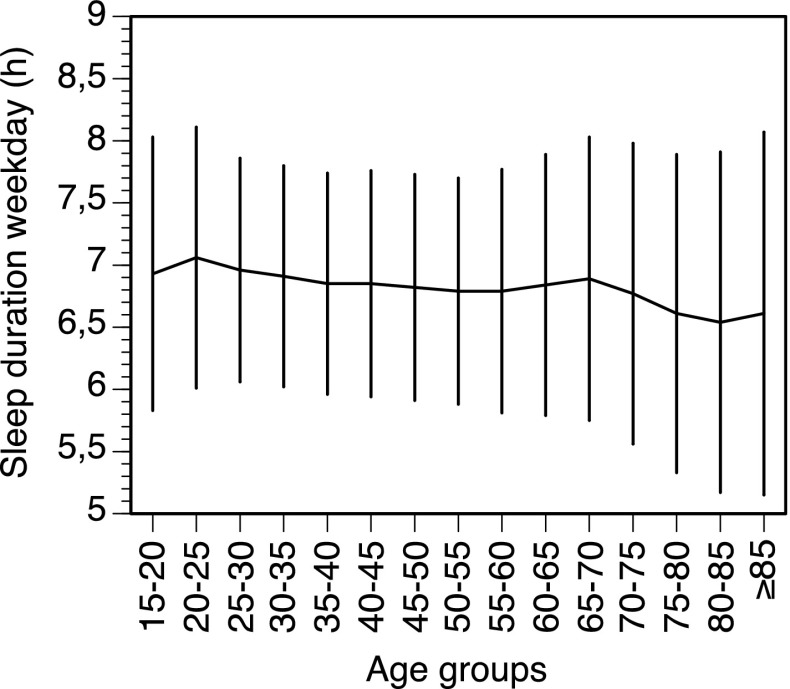
Weekday sleep according to age in 5-year intervals. Mean ± SD

Table 1 shows the distribution of background variables across the groups defined by sleep duration. The participants with short sleep duration were on average older, more frequently men, less educated, and had a higher physical activity level. Participants with a longer sleep duration were less depressed, had a lower intake of coffee, and used less sleep medications. Background data stratified by age are presented as supplementary data (Table S1). Figure 1 shows sleep duration across 5-year age groups. A regression analysis shows that there is a significant reduction in sleep duration as age increases (β = −0.037 h per 10 years (95% CI −0.043 to −0.030), R^2^ = 0.003), but the change is very small.

**Table 1:** Age-standardized baseline characteristics by categories of sleep duration in the Swedish National March Cohort

Characteristic	Sleep duration, hours/day									
	≤5 h		6 h		7 h		≥8 h		Total sample	
	No.	%	No.	%	No.	%	No.	%	No.	%
N	3270		9192		16822		9907		39191	
Years at risk, mean (SD)	12.4 (2.5)		12.7 (2.0)		12.8 (1.8)		12.6 (2.3)		12.7 (2.1)	
Deaths, n	486		761		1255		1046		3548	
Cause specific deaths										
CVD^a^	180	37.0	231	30.3	373	29.7	356	34.0	1140	32.1
Cancer	180	37.0	364	47.8	632	50.4	469	44.8	1645	46.4
Suicide	7	1.5	12	1.6	17	1.3	12	1.2	48	1.3
Respiratory disease	21	4.3	24	3.2	56	4.5	31	3.0	132	3.7
Neurological disease	13	2.7	27	3.6	36	2.9	37	3.5	113	3.2
Other	85	17.5	103	13.5	141	11.2	141	13.5	470	13.3
Age, mean (SD)	55.2 (16.5)		50.5 (15.2)		49.7 (15.0)		50.8 (17.0)		50.6 (13.3)	
Gender, males	1252	38.3	3589	39.0	5970	35.5	3213	32.4	14024	35.8
BMI, mean (SD)	25.2 (4.0)		24.7 (3.6)		24.5 (3.4)		24.6 (3.5)		24.6 (3.5)	
Education										
9 years	1541	42.7	3292	36.6	5761	35.8	3842	38.9	14436	37.2
11–14 years	1060	35.6	3080	34.1	5395	32.2	3274	33.0	12809	33.0
15 years	595	20.8	2650	28.6	5417	31.4	2614	27.4	11276	29.1
Other	34	0.9	69	0.7	103	0.6	76	0.7	282	0.7
Employment										
Employed	1278	58.7	5144	64.2	9336	63.7	3909	56.5	19667	61.8
Unemployed	70	3.1	153	2.0	274	1.9	278	3.6	775	2.4
Retired	1182	24.9	1935	24.1	3355	24.8	2948	27.0	9420	25.2
Sick leave	103	4.3	111	1.5	180	1.3	227	3.2	621	2.0
Other	233	9.0	601	8.2	1111	8.3	923	9.7	2868	8.6
Snoring										
Frequent	307	9.7	744	8.1	1150	6.9	714	7.4	2915	7.5
Infrequent	2395	74.8	7156	78.2	13601	80.9	8038	81.6	31190	80.0
Uncertain	535	15.5	1240	13.7	1992	12.2	1097	11.0	4864	12.5
Good sleep quality	1490	47.3	6790	74.2	14932	89.2	9155	92.7	32367	83.11
Daytime sleepiness	505	17.4	858	9.5	951	5.7	566	5.5	2880	7.4
Work schedule										
Daytime	1300	49.3	5254	57.9	10460	61.8	4973	56.5	21987	58.7
Shiftwork	460	18.6	1253	13.9	1675	9.8	773	8.8	4161	11.1
Other	77	2.9	186	2.3	289	1.9	177	1.8	729	2.0
No work	1035	29.2	1831	25.9	3327	26.5	3080	32.9	9273	28.2
Smoking										
Never	1840	60.7	5153	61.7	10058	64.9	6071	64.9	23122	63.9
Former	791	27.4	2496	28.9	4443	28.0	2453	28.0	10183	28.2
Current	295	11.9	812	9.4	1151	7.1	615	7.1	2873	7.9
Alcohol g/month										
0–23 g	792	22.2	1655	18.4	3215	19.8	2242	22.1	7904	20.0
23–124 g	621	19.8	1774	19.4	3274	19.4	2048	20.8	7717	20.0
124–263 g	602	18.5	1786	19.4	3419	20.2	2000	20.3	7807	20.0
263–503 g	565	17.6	1965	21.3	3502	20.6	1770	18.3	7802	20.0
>503 g	673	21.9	1973	21.5	3361	20.0	1800	18.5	7807	20.0
Self-reported health										
Very good	558	18.5	2278	25.1	4744	28.5	2706	28.1	10286	26.8
Good	1618	51.0	5046	56.2	9295	56.6	5331	55.0	21290	55.7
Average	756	23.5	1442	16.2	2083	12.9	1336	13.7	5617	14.7
Poor	181	6.1	200	2.2	300	1.8	259	2.8	940	2.5
Very poor	26	0.9	28	0.3	32	0.2	44	0.4	130	0.3
Physical activity										
Low	1103	35.1	3620	42.2	6794	42.9	4476	48.7	15993	43.6
Medium	1018	34.2	2956	34.4	5726	35.8	3063	33.0	12763	34.8
High	898	30.7	1984	23.4	3322	21.3	1733	18.3	7937	21.6
Coffee intake, cups/day										
None	314	11.0	972	11.2	2068	12.6	1497	14.2	4851	12.7
1–3	1699	49.7	4489	50.0	8780	53.3	5281	54.2	20249	52.5
4–6	1023	33.0	3119	33.8	5180	30.9	2704	28.8	12026	31.1
≥7	161	6.3	464	5.0	551	3.2	251	2.8	1427	3.7
Nap during day	315	8.1	626	7.1	1069	7.0	1080	10.2	3090	7.9
Hypnotics	951	25.4	1390	15.4	1647	10.3	997	10.0	4985	12.8
Diabetes	128	3.5	222	2.6	333	2.2	251	2.6	934	2.5
Depression	558	18.7	993	10.8	1194	7.0	722	7.4	3467	8.9
Major diseases^b^	677	16.5	1384	15.4	2294	14.6	1632	15.8	5987	15.3

^a^Cardiovascular disease

^b^Diagnosis of cardiovascular disease or cancer before October 1997

**Fig. 2:**
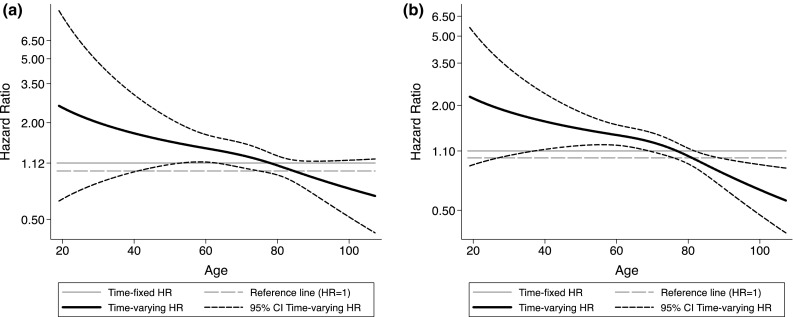
Mortality expressed as time-varying Hazard ratios and 95% confidence intervals for short (**a**) and long (**b**) sleepers versus the 7-h reference group

Table 2 shows the estimated hazard ratios for the relationship between sleep duration and total mortality, both for the entire cohort, and stratified by age. Subjects with short and long sleep duration showed a higher mortality, not statistically significant in the entire cohort. However, sleep duration did not satisfy the PH assumption, which means that the effect of sleep duration on mortality was significantly different across ages [25]. This difference in HR across ages was explored in Fig. 2, which shows that the estimated time varying HRs decreased with increasing age. As expected from the PH assumption test on the exposure, the interaction between sleep duration and age as binary was highly significant (*p* value 0.006) and in the age stratified analyses the PH assumption was satisfied in both age groups. Mortality was significantly higher among individuals below the age of 65 with ≤5 and ≥8 h of sleep compared to 7 h. No significant results were seen for participants ≥65 years. Occupational status, depression, snoring, sleep medication use, napping, sleep quality and sleepiness did not affect the results appreciably and were thus left out of the main analysis (data not shown).

**Table 2:** Sleep duration and mortality: overall and age stratified analysis

Sleep duration, hours/day	Crude				Multivariable adjusted^a^			
	No. of deaths	Person-years	HR	95% CI	No. of deaths	Person-years	HR	95% CI
≤5 h	486	40,469	1.13	1.01, 1.25	337	31,571	1.12	0.99, 1.27
6 h	761	116,938	1.00	0.92, 1.10	564	95,917	0.98	0.88, 1.09
7 h	1255	215,222	1.00	Referent	984	181,815	1.00	Referent
≥8 h	1046	124,483	1.15	1.05, 1.24	805	103,352	1.10	1.00, 1.20
Age <65								
≤5 h	127	28,119	1.45	1.19, 1.77	98	22,409	1.37	1.09, 1.71
6 h	262	96,875	0.99	0.85, 1.15	211	80,596	0.92	0.77, 1.08
7 h	482	181,293	1.00	Referent	396	154,272	1.00	Referent
≥8 h	340	95,764	1.32	1.15, 1.52	270	80,051	1.27	1.08, 1.48
Age ≥65								
≤5 h	359	12,350	1.02	0.90, 1.16	239	9162	1.05	0.90, 1.22
6 h	499	20,063	1.01	0.90, 1.13	353	15,321	1.03	0.90, 1.17
7 h	773	33,930	1.00	Referent	588	27,542	1.00	Referent
≥8 h	706	28,719	1.06	0.96, 1.18	535	23,301	1.01	0.90, 1.14

^a^Hazard ratios were adjusted for age, sex, BMI, smoking status, alcohol consumption, educational level, physical activity and major diseases. Attained age was used as time scale

When stratifying on napping, no significant results were found. However, results stratified both on age and napping showed that long and short sleepers, younger than 65 who did not report napping during the day, retained their original, significant results, while no significant results appeared in the older groups (data not shown).

As a sensitivity analysis we repeated the main analysis (fully adjusted), with the category ≥8 h split into the original 8 and ≥9 h categories, respectively. For the <65 year group, the 8 h category (250 deaths) remained essentially unchanged with HR 1.28 (95% CI 1.09; 1.50), while the ≥9 h category (20 deaths) showed an HR 1.11 (95% CI 0.71; 1.74). No differences in mortality rates were seen in the oldest age group. A sensitivity analysis was also carried out to test the effect of sleep duration on mortality when using 7–8 h (646 deaths) as reference. Among subjects <65 years the results (fully adjusted) showed an increased mortality for the ≤5 h category only, with an HR 1.25 (95% CI 1.01, 1.55). Sleeping 6 h showed a HR of 0.84 (95% CI 0.72–0.98), while no increased mortality was detected for the ≥9 h category (HR 1.01, 95% CI 0.65, 1.58). No significant results were observed for the same analysis in the ≥65-year group.

For the relationship between sleep duration and cause-specific mortality, and its link to age, we did not observe any significant pattern for the overall group or among subjects above the age of 65 years. Table 3 shows the results from cause-specific mortality due to CVD and cancer exclusively for subjects <65 years of age. CVD mortality was significantly increased among individuals with long sleep duration. For cancer, no evidence of significant associations was found after full adjustment.

**Table 3:** Sleep duration and cause specific mortality for subjects < 65 years

Sleep duration, hours/day	<65 years							
	Crude				Multivariable adjusted^a^			
	No. of deaths	Person-years	HR	95% CI	No. of deaths	Person-years	HR	95% CI
CVD mortality								
≤5 h	22	28,119	1.40	0.87, 2.24	18	22,409	1.41	0.83, 2.37
6 h	44	96,875	0.96	0.66, 1.38	33	80,596	0.84	0.56, 1.28
7 h	83	181,293	1.00	Referent	66	154,272	1.00	Referent
≥8 h	66	95,764	1.45	1.05, 2.01	55	80,051	1.54	1.07, 2.21
Cancer mortality								
≤5 h	73	28,119	1.25	0.97, 1.61	56	22,409	1.13	0.83, 1.53
6 h	163	96,875	0.92	0.76, 1.11	132	80,596	0.86	0.70, 1.07
7 h	323	181,293	1.00	Referent	271	154,272	1.00	Referent
≥8 h	212	95,764	1.25	1.05, 1.48	168	80,051	1.16	0.94, 1.42

^a^ Hazard ratios were adjusted for age, sex, BMI, smoking status, alcohol consumption, educational level, physical activity and major diseases. Attained age was used as time scale

To explore the age effect further, time varying HRs are graphically reported in Fig. 2. The HRs for both short and long sleep present a decreasing pattern with respect to age, being greater than 2 in the youngest and equal to 1 or even below, in the oldest. The age span of significant excessive mortality for short sleepers was 41.0–74.5 years. This represents 70.9% of the sample, with 19.1% below and 10.0% of the sample above the span. For long sleepers the corresponding age span was 27.0–75.5 years, while a reduced mortality was observed beyond 88.6 years. The age span with higher mortality represents 82.9% of the sample, with 13.3% below and 3.7% of the sample above the span, but below 88.6 years. Only 0.1% of the long sleepers was above this age.

To complement the results from the previous analysis, multivariable Cox regressions were run by stratifying on two different cut points (45 and 70 years). In both cases significant results were observed only in the category including the younger subjects. In those below 45 years the HR was 2.45 (95% CI 1.19–5.04) for short sleep (≤5 h) and 2.10 (95% CI 1.32–3.34) for long sleep. In those below 70 years the corresponding HR values for short and long sleep were 1.38 (95% CI 1.16–1.65) and 1.20 (95% CI 1.06–1.36), respectively. Finally, we also used the standard 60-year cut-point, given that it has been used in the few studies that have been carried out on age effects. This yielded results very similar to those with the cut-point at 65 years. See Table S2.

The influence of age was also examined by using Laplace regression to estimate different percentiles of the distribution of age at death. Table 4 shows that at the 25th percentile of age at death, short and long sleepers died 8.5 (95% CI −16.7; −0.4) and 6.6 months (95% CI −12.4; −0.7) earlier, respectively. Smaller estimates and no significant relationships were seen for the other age percentiles, denoting a stronger effect of sleeping among subjects who died younger.

**Table 4:** Differences in months in age at death across levels of sleep duration in the Swedish National March Cohort

Sleep duration, hours/day	No. of deaths	Person-years	25th PD, months		50th PD, months		75th PD, months	
			PD	95% CI	PD	95% CI	PD	95% CI
Model 1^a^								
≤5 h	486	40,469	−8.8	−15.3, −2.3	−6.7	−12.9, −0.5	−7.0	−12.8, −1.2
6 h	761	116,938	−0.5	−5.9, 4.9	−0.3	−5.5, 4.8	−0.9	−6.0, 4.1
7 h	1255	215,222	0	Referent	0	Referent	0	Referent
≥8 h	1046	124,483	−9.5	−14.7, −4.4	−6.9	−11.5, −2.2	−6.9	−11.5, −2.2
Model 2^b^								
≤5 h	337	31,571	−8.5	−16.7, −0.4	−6.2	−13.2, 0.9	−6.5	−13.4, 0.4
6 h	564	95,917	1.4	−4.8, 7.7	1.7	−4.2, 7.6	0.2	−5.5, 5.8
7 h	984	181,815	0	Referent	0	Referent	0	Referent
≥8 h	805	103,352	−6.6	−12.4, −0.7	−5.3	−10.6, 0.1	−3.6	−9.1, 1.9

*PD* percentile difference

^a^Estimates were obtained by fitting a Laplace regression on the 25th, 50th, and the 75th percentiles of age at death adjusted for age at baseline

^b^The model was further adjusted for sex, body mass index, smoking status, alcohol consumption, educational level, physical activity and major diseases

After excluding cases occurring in the first 2 years of follow-up, only minor changes in the results were observed. For subjects <65 years we confirmed a statistically significant association between sleep duration and mortality (for ≤5 h of sleep, HR 1.31, 95% CI 1.03–1.65; for ≥8 h of sleep, HR 1.23, 95% CI 1.05–1.45). For subjects aged 65 or older no statistically significant association was detected.

We lacked information on sleep duration for 2456 subjects. These were older, less educated and with more health problems. By comparing the adjusted survival curves of individuals with and without information on the exposure, no difference in survival was observed. As a result, when eliminating subjects with missing values on sleep duration from the analysis, the true survival of the cohort ought not be overestimated.

The results obtained using the imputed dataset are similar to those after excluding subjects with missing values on covariates: with 7 h as reference the estimates among the youngest (<65 years) were 1.38 (95% CI 1.13–1.68) for short sleep and 1.29 (95% CI 1.12–1.48) for long sleep. No significant association was detected among the oldest (≥65 years) (for ≤5 h HR 1.07, 95% CI 0.94–1.22; for ≥8 h, HR 1.02, 95% CI 0.92–1.13).

## Discussion

In this large, prospective cohort, individuals younger than 65 years, who at baseline reported either short or long sleep duration, showed an increased mortality. In those aged 65 or older, there was no significant association between sleep duration and mortality. The effect of sleep duration on mortality was highest among young individuals and decreased with increasing age. The quantile regression analysis supported this finding.

The U-shaped relation between sleep duration and mortality essentially agrees with previous meta analyses [1–3, 10], except for Liu et al [10], who found a J-shaped one.

Still, the present results deviate from the meta-analyses of Cappuccio [1] and Silva [3] in which, in contrast to our findings, a clear effect of sleep duration in individuals ≥60 years was shown. This relationship was also supported by three original studies [7–9], whereas Liu et al. [10] in their meta-analysis did find an increased effect for long sleep but not for short sleep in individuals >65 years.

However, if the decrease in HR with age is a correct conclusion, then the strength and the significance of the effect of sleep duration on mortality will depend on the proportion of older participants in the study. The larger the proportion, the less the likelihood would be of a significant result. Thus, varying proportions of older participants in different studies could contribute to the variability in conclusions found in the literature. In the present cohort, the proportion of individuals ≥75 years (at which point the HR was no longer significantly above unity) among participants aged 65 or older was 19%. Corresponding proportions are not available from other studies.

The observed decline in the hazard ratio as a function of age is a new finding and there is no clear explanation for this decreasing pattern. However, the spectrum of causes of death changes with age and this may dilute any effect of sleep duration on mortality in higher age groups. Another explanation of the lack of effect of sleep duration in higher age groups may be that older individuals represent a survivor population that may be particularly resilient to negative health effects. A third explanation may be the effects of being retired on the precision of estimations of sleep duration. Not having to rise at a certain time to go to work could deprive the individual of an important time reference. This loss of reference may reduce the precision of estimates of sleep duration and thus result in misclassification of the exposure and in a weaker association between sleep duration and mortality. This issue does not, however, seem to have been formally investigated. It should be emphasized that there was very little change in sleep duration across age groups (1.2 min per year), suggesting that mean sleep duration is unlikely to be a part of the loss of relationship between sleep duration and mortality with increasing age.

Our original hypothesis was that the decreased sleep duration with age [5] would result in a stronger link to mortality for long sleepers, because long sleep would deviate from the norm. Similarly, short sleep in younger individuals was expected to lead to increased mortality, again because short sleep would deviate from the age norm. These hypotheses were not confirmed; rather it seems that long and short sleep have similar relationships with mortality regardless of age group and that the association disappears in higher age groups. In fact, inspection of Fig. 2 shows that mortality hazard ratio falls below unity above the age of approximately 87 years. This might be a spurious result based on rather few deaths. It might possibly also indicate that with increasing age, the ability to produce long sleep might be an indication of good health. Addressing this interesting age issue will require a larger sample in higher age groups than what is available in the present study.

The explanation of the U-shaped relationship between sleep duration and mortality has been discussed in most previous work in this area. Conditions like comorbidities, BMI, alcohol consumption, education, employment status, work schedules, snoring, physical activity, napping, use of hypnotics, depression, sleep quality, sleepiness and snoring were considered and adjusted for in the present study, or tested separately, and should not have affected outcomes. Grandner et al. [6] suggested that sleep loss might have similar effects to those caused by low grade inflammation and that increased levels of ghrelin, increased lipid levels, and insulin resistance could be involved in this process. The mechanism involved in the association between long sleep and mortality still defies speculation, apart from the possibility of residual confounding or undetected diseases.

The reason for the lack of effect of napping may be that napping was rarely reported in the present cohort, particularly in younger individuals. Furthermore, we did not collect information on the duration of the naps, which is a limitation. Still, the few previous original studies on napping do not provide any clear conclusions [11, 12, 26]. Leaving out napping resulted in a lack of significant effect for short sleep in the meta regression of Liu et al. [10]. Still, the role of napping in relation to mortality remains unclear.

The sensitivity analysis using different cut-offs for sleep duration showed that long sleep defined as ≥9 h yielded a non-significant hazard ratio in both age groups. The reason for this is not clear, but the number of deaths in subjects below the age of 65 in this sleep category was very low (20), as was the number of person-years (6379). This invites chance effects – only a few more deaths would have increased the estimate for the hazard ratio, albeit with large confidence intervals. Short sleep (≤5 h), the second smallest category, contained 5 times as many deaths and 3.5 times as many person-years.

Using 7–8 h of sleep as reference and defining long sleep as ≥9 h had two consequences. Firstly, long sleep duration was not significant and the point estimate became closer to one. It’s likely that the higher mortality in the 8 h category raised mortality above the level of that of 7 h of sleep, thus reducing the difference between the reference and the ≥9 h category. Secondly, for the same reason, the 6 h category showed a significant HR below 1. It was also associated with the lowest mortality in the original analysis with 7 h as reference, but the estimated HR, despite being below 1, was not significant. These results might suggest that, in the present cohort, the optimal sleep duration with respect of mortality is 6 h, rather than 7 h.

The strengths of the present study are its size, the length of the follow-up, the ability to directly link baseline information with mortality registers through the national registration number and the high quality of the data. However, as with virtually all studies of sleep duration, it relies on self-reported information. This is clearly a weakness, but one which is shared with many other exposure variables in the field of lifestyle epidemiology. Though, it has been found a strong correlation between the average sleep measured through 7-day diaries and that obtained from one sleep-duration question [27] with small systematic bias caused by a tendency of overestimation [28].

Similarly, most confounders are also self-reported. One confounder not included was sleep apnea, however, we did adjust for snoring, which is probably a reasonable proxy. Another limitation of our study concerns the measurement of the exposure only at baseline, thus potential changes in sleep duration during follow-up would go undetected.

Major disease is generally seen as the most crucial variable to adjust for in studies of sleep duration and mortality. This was done in the present study, however, precursors of major diseases (such as increased levels of lipids or blood sugar) could also play a role, but this information was not available. Kurina et al. [4] have remarked that major diseases, not yet diagnosed, may be an important contributor to unreliable results. To minimize the risk of reverse confounding, we excluded cases occurring in the two first years of follow-up, but the results remained virtually unchanged.

Kurina et al. [4] also pointed out that an important issue is the subjective estimate of sleep duration. This may be a problem, but it does not seem likely that individuals who are healthy at baseline would misclassify their sleep duration to a higher degree based on future mortality. Van den Berg et al [29], however, found that subjective estimates of sleep duration differed more from objective ones depending on sleep quality, gender, poor cognitive function and functional disability. We adjusted for the two first, but the two latter variables may have affected the estimates of sleep duration, particularly among the older participants. This could have contributed to the lack of significant association between sleep duration and mortality in the older group. It should be emphasized that in the present study, as in most other studies [30], we analyzed weekday sleep, which means that sleep was restricted by work for most individuals. Possibly, weekend sleep may also be of interest for studying the relationship between sleep duration and mortality.

In conclusion, the present study showed that increasing age reduces the relationship between sleep duration and mortality and that the relationship is particularly strong in younger individuals.

## Electronic supplementary material

Below is the link to the electronic supplementary material.

Supplementary material 1 (DOCX 285 kb)

## References

[1] Cappuccio FP, D’Elia L, Strazzullo P (2010). Miller MA Sleep duration and all-cause mortality: a systematic review and meta-analysis of prospective studies. Sleep.

[2] Gallicchio L, Kalesan B (2009). Sleep duration and mortality: a systematic review and meta-analysis. J Sleep Res.

[3] Silva AA, Mello RG, Schaan CW, Fuchs FD, Redline S (2016). Fuchs SC Sleep duration and mortality in the elderly: a systematic review with meta-analysis. BMJ Open.

[4] Kurina LM, McClintock MK, Chen JH, Waite LJ, Thisted RA (2013). Lauderdale DS Sleep duration and all-cause mortality: a critical review of measurement and associations. Ann Epidemiol.

[5] Ohayon MM, Carskadon MA, Guilleminault C (2004). Vitiello MV Meta-analysis of quantitative sleep parameters from childhood to old age in healthy individuals: developing normative sleep values across the human lifespan. Sleep.

[6] Grandner MA, Patel NP, Gehrman PR, Perlis ML (2010). Pack AI Problems associated with short sleep: bridging the gap between laboratory and epidemiological studies. Sleep Med Rev.

[7] Yeo Y, Ma SH, Park SK (2013). A prospective cohort study on the relationship of sleep duration with all-cause and disease-specific mortality in the Korean Multi-center Cancer Cohort study. J Prev Med Public Health.

[8] Gangwisch JE, Heymsfield SB, Boden-Albala B (2008). Sleep duration associated with mortality in elderly, but not middle-aged, adults in a large US sample. Sleep.

[9] Hublin C, Partinen M, Koskenvuo M, Kaprio J (2007). Sleep and mortality: a population-based 22-year follow-up study. Sleep.

[10] Liu TZ, Xu C, Rota M et al. Sleep duration and risk of all-cause mortality: a flexible, non-linear, meta-regression of 40 prospective cohort studies. Sleep Med Rev 2016; (**in press**). 10.1016/j.smrv.2016.02.005 27067616

[11] Lan TY, Lan TH, Wen CP, Lin YH, Chuang YL (2007). Nighttime sleep, Chinese afternoon nap, and mortality in the elderly. Sleep.

[12] Cohen-Mansfield J, Perach R (2012). Sleep duration, nap habits, and mortality in older persons. Sleep.

[13] Lagerros YT, Bellocco R, Adami HO, Nyren O (2009). Measures of physical activity and their correlates: the Swedish National March Cohort. Eur J Epidemiol.

[14] Trolle Lagerros Y, Hantikainen E, Ye W et al. Cohort profile: the Swedish National March Cohort (SNMC). Int J Epidemiol. 2016 (**accepted**). 10.1093/ije/dyw193 27649800

[15] Akerstedt T, Ingre M, Broman JE, Kecklund G (2008). Disturbed sleep in shift workers, day workers, and insomniacs. Chronobiol Int.

[16] Kripke DF, Garfinkel L, Wingard DL, Klauber MR, Marler MR (2002). Mortality associated with sleep duration and insomnia. Arch Gen Psychiatry.

[17] Pearl J (2009). Causality: models, reasoning, and inference.

[18] Textor J, Hardt J, Knuppel S (2011). DAGitty: a graphical tool for analyzing causal diagrams. Epidemiology.

[19] Ainsworth BE, Haskell WL, Herrmann SD (2011). Compendium of physical activities: a second update of codes and MET values. Med Sci Sports Exerc.

[20] Heinzl H, Kaider A (1997). Gaining more flexibility in Cox proportional hazards regression models with cubic spline functions. Comput Methods Programs Biomed.

[21] Harrell F (2015). Regression modeling strategies: with applications to linear models, logistic and ordinal regression, and survival analysis.

[22] Bellavia A, Discacciati A, Bottai M, Wolk A, Orsini N (2015). Using laplace regression to model and predict percentiles of age at death when age is the primary time scale. Am J Epidemiol.

[23] Lambert PC (2009). Royston P Further development of flexible parametric models for survival analysis. Stata J.

[24] van Buuren S, Boshuizen HC, Knook DL (1999). Multiple imputation of missing blood pressure covariates in survival analysis. Stat Med.

[25] Lamarca R, Alonso J, Gomez G, Munoz A (1998). Left-truncated data with age as time scale: an alternative for survival analysis in the elderly population. J Gerontol A Biol Sci Med Sci.

[26] Stone KL, Ewing SK, Ancoli-Israel S (2009). Self-reported sleep and nap habits and risk of mortality in a large cohort of older women. J Am Geriatr Soc.

[27] Patel SR, Ayas NT, Malhotra MR (2004). A prospective study of sleep duration and mortality risk. Sleep.

[28] Lauderdale DS, Knutson KL, Yan LL, Liu K, Rathouz PJ (2008). Self-reported and measured sleep duration: how similar are they?. Epidemiology.

[29] Van Den Berg JF, van Rooij FJ, Vos H (2008). Disagreement between subjective and actigraphic measures of sleep duration in a population-based study of elderly persons. J Sleep Res.

[30] Lauderdale SD (2014). Survey questions about sleep duration: does asking separately about weekdays and weekends matter?. Behav Sleep Med.

