# Supplementary material for: Sleep duration, mortality and the influence of age
Source: Eur J Epidemiol. 2017 Aug 30;32(10):881–91. doi: 10.1007/s10654-017-0297-0 (PMC5680380; doi:10.1007/s10654-017-0297-0)
Supplement: Supplementary file 1 — Supplementary material 1 (DOCX 285 kb) [file 10654_2017_297_MOESM1_ESM.docx]

FOR SUPPLEMENT

Table S1. Age standardized baseline characteristics by categories of age in the Swedish National March Cohort

| **Characteristics** | **< 65 years** | |  | ≥ **65 years** | | |  |
| --- | --- | --- | --- | --- | --- | --- | --- |
|  | **No.** | **%** |  | **No.** | | **%** |  |
| N | 31,102 79.4 | |  | 8,089 20.6 | | |  |
| Years at risk, mean | 12.9 | |  | 11.8 | | |  |
| Deaths, n | 1,211 | |  | 2,337 | | |  |
| Cause specific deaths  CVD^1^  Cancer  Suicide  Respiratory disease  Neurological disease  Other | 215  771  35  29  31  130 | 17.7  63.7  2.9  2.4  2.6  10.7 |  | 925  874  13  103  82  340 | 39.6  37.4  0.6  4.4  3.5  14.5 | |  |
| Sleep duration  ≤ 5 hours  6 hours  7 hours  ≥ 8 hours | 2,198  7,486  13,969  7,449 | 7.0  24.1  44.9  24.0 |  | 1,072  1,706  2,853  2,458 | 13.3  21.1  35.3  24.8 | |  |
| Age, mean | 45.3 | |  | 71.0 | | |  |
| Gender, males | 10,145 | 32.6 |  | 3,879 | 47.9 | |  |
| BMI, mean | 24.5 | |  | 25.0 | | |  |
| Education  9 years  11-14 years  15 years  Other | 9,526  11,138  9,988  180 | 30.9  36.1  32.4  0.6 |  | 4,910  1,671  1,288  102 | | 61.6  21.0  16.1  1.3 |  |
| Employment  Employed  Unemployed  Retired  Sick leave  Other | 19,589  775  1,542  618  2,827 | 77.3  3.0  6.1  2.4  11.2 |  | 78  0  7,878  3  41 | | 1.0  0.0  98.4  0.1  0.5 |  |
| Work schedule  Daytime  Shiftwork  Other  No work | 21,377  4,116  609  3,678 | 71.8  13.8  2.0  12.4 |  | 610  45  120  5,595 | | 9.6  0.7  1.9  87.8 |  |
| Snoring  Frequent  Infrequent  Uncertain | 2,215  25,256  3,511 | 7.2  81.5  11.3 |  | 700  5,934  1,353 | | 8.8  74.3  16.9 |  |
| Smoking  Never  Former  Current | 18,011  8,237  2,637 | 62.4  28.5  9.1 |  | 5,111  1,947  235 | | 70.1  26.7  3.2 |  |
| Alcohol g/month  0-23g  23-124g  124-263g  263-503g  >503g | 5,486  6,431  6,270  6,459  6,359 | 17.7  20.8  20.2  20.8  20.5 |  | 2,418  1,286  1,537  1,343  1,448 | | 30.1  16.0  19.2  16.7  18.0 |  |
| Self-reported Health  Very good  Good  Average  Poor  Very poor | 8,673  16,763  4,153  772  113 | 28.5  55.0  13.6  2.5  0.4 |  | 1,613  4,527  1,464  168  17 | | 20.7  58.1  18.8  2.2  0.2 |  |
| Physical activity  Low  Medium  High | 12,641  10,363  6,294 | 43.1  35.4  21.5 |  | 3,352  2,400  1,643 | | 45.3  32.5  22.2 |  |
| Coffee intake, cups/day  None  1-3  4-6  ≥ 7 | 4,416  15,282  9,586  1,315 | 14.450.0  31.3  4.3 |  | 435  4,967  2,440  112 | | 5.5  62.4  30.7  1.4 |  |
| Nap during day | 1,571 | 5.1 |  | 1,519 | | 18.9 |  |
| Hypnotics | 3,054 | 9.8 |  | 1,931 | | 24.0 |  |
| Diabetes | 557 | 1.9 |  | 377 | | 5.1 |  |
| Depression | 3,027 | 9.8 |  | 440 | | 5.6 |  |
| Major diseases | 3,273 | 10.5 |  | 2,714 | | 33.6 |  |

^1^ Cardiovascular disease

^2^ Diagnosis of cardiovascular disease or cancer before October 1997

Table S2. Sleep duration and mortality: age stratified analysis using three different cut-offs.

| **Sleep duration, hours/day** | **No. of deaths** | **Person-years** | **HR** | **95% CI** |  | **No. of deaths** | **Person-years** | **HR** | **95% CI** |
| --- | --- | --- | --- | --- | --- | --- | --- | --- | --- |
|  | **Age < 45** | | | |  | **Age ≥ 45** | | | |
| ≤ 5 h | 10 | 8,293 | 2.45 | 1.19, 5.04 |  | 327 | 23,278 | 1.09 | 0.96, 1.24 |
| 6 h | 25 | 33,421 | 1.35 | 0.80, 2.26 |  | 539 | 62,496 | 0.97 | 0.87, 1.08 |
| 7 h | 36 | 68,092 | 1.00 | Referent |  | 948 | 113,723 | 1.00 | Referent |
| ≥ 8 h | 37 | 38,425 | 2.10 | 1.32, 3.34 |  | 768 | 64,927 | 1.07 | 0.97, 1.17 |
|  | **Age < 60** | | | |  | **Age ≥ 60** | | | |
| ≤ 5 h | 59 | 18,613 | 1.39 | 1.05, 1.85 |  | 278 | 12,958 | 1.08 | 0.94, 1.24 |
| 6 h | 140 | 70,864 | 0.91 | 0.74, 1.12 |  | 424 | 25,053 | 1.01 | 0.90, 1.14 |
| 7 h | 262 | 136,550 | 1.00 | Referent |  | 722 | 45,264 | 1.00 | Referent |
| ≥ 8 h | 155 | 68,720 | 1.27 | 1.04, 1.55 |  | 650 | 34,632 | 1.05 | 0.94,1.17 |
|  | **Age < 70** | | | |  | **Age ≥ 70** | | | |
| ≤ 5 h | 163 | 26,169 | 1.38 | 1.16, 1.65 |  | 174 | 5,402 | 0.96 | 0.80, 1.15 |
| 6 h | 314 | 88,328 | 0.96 | 0.84, 1.10 |  | 250 | 7,589 | 1.03 | 0.88, 1.21 |
| 7 h | 594 | 169,573 | 1.00 | Referent |  | 390 | 12,242 | 1.00 | Referent |
| ≥ 8 h | 457 | 92,697 | 1.20 | 1.06, 1.36 |  | 348 | 10,655 | 0.97 | 0.84, 1.12 |

Figure S1. Directed acyclic graph. Biasing paths are indicated by red arrows, causal path are indicated by green arrows. Ancestors of outcome are drawn in blue, ancestors of exposure and outcome are drawn in red.


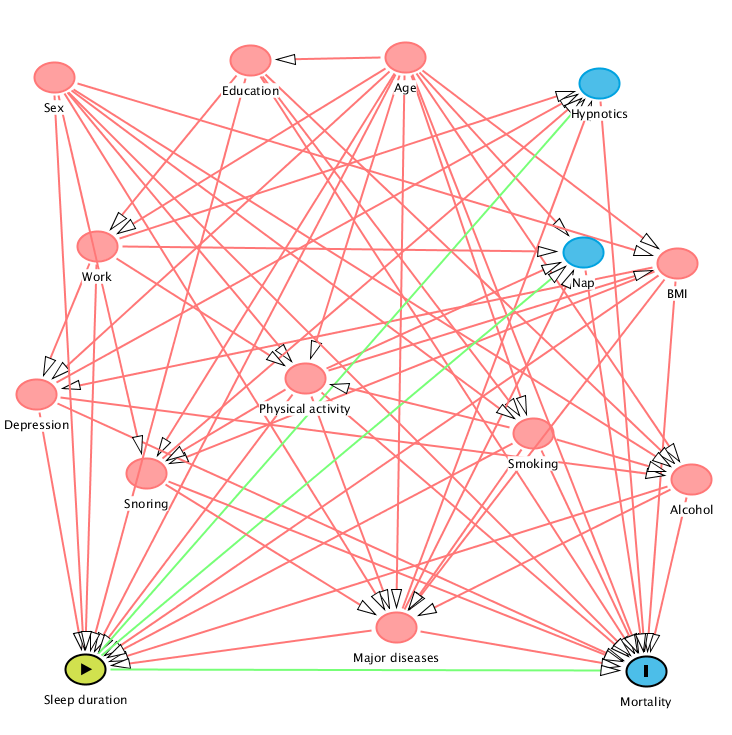


Minimal sufficient adjustment sets for estimating the total effect of sleep duration on mortality: Age, Alcohol, BMI, Depression, Education, Major diseases, Physical activity, Sex, Smoking, Work.
